# Mechanical Ventilation Boot Camp: A Simulation-Based Pilot Study

**DOI:** 10.1155/2016/4670672

**Published:** 2016-02-01

**Authors:** Jennifer Yee, Charles Fuenning, Richard George, Rana Hejal, Nhi Haines, Diane Dunn, M. David Gothard, Rami A. Ahmed

**Affiliations:** ^1^Summa Health System Akron City Hospital, Akron, OH 44304, USA; ^2^Northeast Ohio Medical University, Rootstown, OH 44272, USA; ^3^Western Reserve Hospital, Cuyahoga Falls, OH 44223, USA; ^4^Akron Children's Hospital, Akron, OH 44308, USA; ^5^BIOSTATS, Inc., East Canton, OH 44730, USA

## Abstract

*Objectives*. Management of mechanically ventilated patients may pose a challenge to novice residents, many of which may not have received formal dedicated critical care instruction prior to starting their residency training. There is a paucity of data regarding simulation and mechanical ventilation training in the medical education literature. The purpose of this study was to develop a curriculum to educate first-year residents on addressing and troubleshooting ventilator alarms.* Methods*. Prospective evaluation was conducted of seventeen residents undergoing a twelve-hour three-day curriculum. Residents were assessed using a predetermined critical action checklist for each case, as well as pre- and postcurriculum multiple-choice cognitive knowledge questionnaires and confidence surveys.* Results*. Significant improvements in cognitive knowledge, critical actions, and self-reported confidence were demonstrated. The mean change in test score from before to after intervention was +26.8%, and a median score increase of 25% was noted. The ARDS and the mucus plugging cases had statistically significant improvements in critical actions, *p* < 0.001. A mean increase in self-reported confidence was realized (1.55 to 3.64), *p* = 0.049.* Conclusions*. A three-day simulation curriculum for residents was effective in increasing competency, knowledge, and confidence with ventilator management.

## 1. Introduction

Management of patients requiring mechanical ventilation is complex and poses a substantial patient safety risk for novice practitioners. Understanding the patient-ventilator interaction is essential to minimize the risk of iatrogenic barotrauma, ventilator-associated pneumonia (VAP), and death [1]. A lack of dedicated formal instruction on ventilator modes and patient-ventilator interaction leads to varying levels of comfort and ability to manage these patients. National work-hour restrictions, medicolegal concerns, and the increasing complexity and volume of critical care patients in the United States significantly limit the time allowed for learning key concepts and developing autonomy with mechanical ventilator management during ICU (Intensive Care Unit) rotations [2].

Simulation is effective as a training methodology within medical education [3, 4], including the development and refinement of critical care skills [5, 6], invasive procedures [7–11], and crisis resource management [12, 13]. Residents are evaluated on their clinical development by assessing their proficiency in the aforementioned skill sets. The Accreditation Council of Graduate Medical Education (ACGME) has established milestone guidelines to help residency programs standardize performance expectations, facilitate feedback for development, and identify areas needing further guidance. The ACGME mandates the use of simulation in emergency medicine, surgery, and internal medicine training [14–16]. Singer et al. demonstrated that first-year residents who underwent simulation-based education outperformed traditionally taught third-year residents in critical care medicine topics [17]. Frengley et al. reported that teamwork in critical care teams could be improved after simulated-based training intervention [18]. However, one area lacking sufficient study in the medical education literature is formal training in the management of mechanically ventilated patients. Even in today's medicolegal environment where “learning on patients” is no longer acceptable, many intensivists report a lack of standardization to their own mechanical ventilation training.

The aim of this study was to develop and pilot test an interactive mechanical ventilation boot camp curriculum for first-year residents in surgery and emergency medicine. The objective of the training was to familiarize learners with common modes of ventilation, common etiologies of ventilator alarms and subsequent management strategies, and high-risk low-frequency presentations. We hypothesize that the use of a standardized curriculum would improve cognitive knowledge, performance, and participant confidence with ventilator management.

## 2. Materials and Methods

### 2.1. Study Location and Equipment

**Figure 1:**
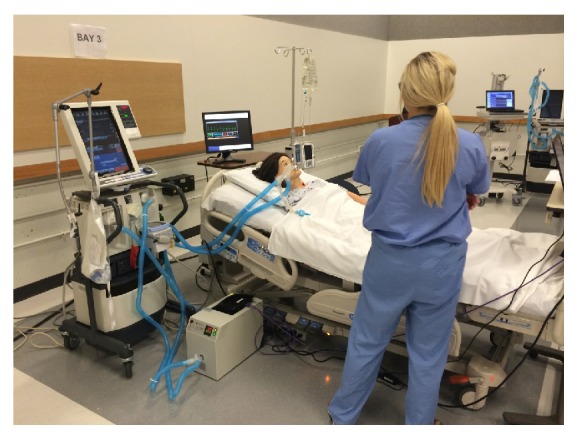
Mechanical ventilation training and testing environment.

The study was performed at a tertiary-care university-affiliated teaching hospital simulation lab during July of 2015. Human-patient simulators in ICU beds were connected to ASL 5000 Breathing Simulators (IngMar Medical). Each of the five human-patient simulators were intubated and connected to a mechanical ventilator (Covidien Puritan Bennett*™*840). ASL 5000 Lung simulators were used to adjust the pulmonary mechanics (lung compliance, airway resistance, respiratory rate, and tidal volume) in real-time based on the actions of the resident. Each bay had a simulated patient monitor that demonstrated the patients' vital signs, portable chest X-rays, electrocardiograms, and arterial blood gas measurements (if requested), as shown in Figure 1. This was a quality assurance project that did not meet the definition of human subject research. It was exempt from institutional review board review.

### 2.2. Curriculum Development and Outline

A three-day pilot boot camp curriculum was developed to educate first-year residents on the management of patients requiring intubation and mechanical ventilatory support. The boot camp program required approximately 65 hours of initial preparation from the simulation and ICU faculty for the development of the curriculum, surveys, questionnaires, and simulation cases with accompanying didactic postsimulation lectures. After initial curriculum development, all participating staff performed a rehearsal of the boot camp to assure that simulation cases were executed without difficulties and all necessary equipment was available and working appropriately. This required an additional 3 hours. The boot camp was scheduled for 12 hours over three days. Overall, total preparation and execution of the curriculum took approximately 80 hours.

The curriculum consisted of 4 parts (as shown in* Three-Day Mechanical Ventilation Simulation Curriculum*): preintervention evaluation, independent study, the intervention phase, and postintervention evaluation. Cognitive tests, critical action checklists, and confidence surveys were used to primarily assess the residents. The entire curriculum took participants approximately twelve hours to complete. The cognitive tests, critical action checklists, and confidence surveys were identical for both pre- and postintervention evaluation.

*Three-Day Mechanical Ventilation Simulation Curriculum*

 Day 1 (Pretesting Evaluation):
   Pretest confidence survey (5 minutes).
   Pretest cognitive multiple-choice exam (25 minutes).
   Cases and evaluation by critical actions checklist (10 minutes each for 30 minutes total):
     ARDS.
     Complete lung atelectasis secondary to mucus plugging.
     Pneumothorax in a mechanically ventilated patient.
   Received supplemental readings.

Independent study (estimated 4 hours of reading material provided for asynchronous education).

 Day 2 (Curriculum and Educational Intervention):
   Case structure:
     Two-three residents participated in the case (10 minutes).
     Evaluation by critical actions checklist (evaluated during case).
     Bedside debriefing by intensivists (20 minutes).
     Review of provided didactic PowerPoint (15 minutes).
   Pathology reviewed:
     ARDS.
     Complete lung atelectasis secondary to mucus plugging.
     Altered mental status secondary to overdose.
     Pneumothorax in a mechanically ventilated patient.
     Dynamic hyperinflation.
 Day 3 (Posttesting Evaluation):
   Posttest confidence survey (5 minutes).
   Posttest cognitive multiple-choice exam (25 minutes).
   Cases and evaluation by critical actions checklist (ten minutes each for 30 minutes total):
     ARDS.
     Complete lung atelectasis secondary to mucus plugging.
     Pneumothorax in a mechanically ventilated patient.

### 2.3. Participants, Faculty, and Staff

First-year residents from three residency programs were invited to participate in the curriculum, which was conducted in July. Six intensivists were present for debriefing based on availability, as well as one emergency medicine attending physician and two respiratory therapists from a local teaching hospital. When present, the faculty debriefers were assigned to one station for the day and did not rotate through different cases. Each station required a simulation technician for all three days of curriculum.

### 2.4. Preintervention Evaluation

The preintervention stage assessed baseline knowledge and confidence on 3 simulated high-risk mechanical ventilation scenarios, including acute respiratory distress syndrome, complete lung atelectasis secondary to obstruction from a mucus plug (hereby referred to as mucus plugging), and pneumothorax. Participants filled out a pretest 12-question confidence survey (as shown in* Confidence Survey*) and a cognitive 20-question multiple-choice test. Each station accommodated one resident at a time. Residents transitioned through the stations based on availability. One intensivist was based at a station per day. The intensivists rated participants' performance using a predetermined checklist of critical actions. The critical actions assessed for the mucus plugging case are given herein after. Feedback was not given to participants at any time in the preintervention phase.

*Confidence Survey*

Four digit identifying code:

Surgery or Emergency Medicine

1. very uncomfortable
2. somewhat uncomfortable
3. neutral
4. somewhat comfortable
5. very comfortable

 

1. How comfortable do you feel distinguishing between the different modes of ventilation?
2. How comfortable do you feel initially choosing a mode of ventilation?
3. How comfortable do you feel switching between different modes of ventilation?
4. How comfortable do you feel weaning mechanical ventilation?
5. How comfortable do you feel addressing an alarming ventilator?
6. How comfortable do you feel identifying causes of an elevated peak airway pressure?
7. How comfortable do you feel identifying causes of an elevated plateau pressure?
8. How comfortable do you feel managing the mechanical ventilation of a patient with ARDS?
9. How comfortable do you feel managing the mechanical ventilation of a patient with decompensated CHF?
10. How comfortable do you feel managing the mechanical ventilation of a patient with an acute exacerbation of COPD?
11. How comfortable do you feel managing the mechanical ventilation of a patient with an acute exacerbation of asthma?
12. How comfortable do you feel managing the mechanical ventilation of a patient with a traumatic brain injury?

*Critical Actions Checklist for Mucus Plugging Case*

1. Identifying mucus plugging
  □ Yes
  □ No
2. Identifying increased airway resistance with high peak pressure
  □ Yes
  □ No
3. Actions (suctioning, bronchoscopy, bronchodilators)
  - Must include either suctioning or bronchoscopy
  - Yes
  - No
4. To increase FiO_2_ to maintain oxygenation
  □ Yes
  □ No

(5) To decrease tidal volume while increasing rate to maintain minute ventilation
  □ Yes
  □ No
(6) Reevaluate the patient after vent changes
  □ Yes
  □ No
(7) Documenting ventilator setting changes
  □ Yes
  □ No

Immediately after initial testing, residents were provided with reading material. This asynchronous material consisted of approximately four hours of reading and included selections taken from book chapters, a review article, and a primer reviewing basic mechanical ventilation principles created by one of the intensivists.

### 2.5. Educational Intervention

The intervention phase was comprised of five 45-minute scenarios and took place six days after pretesting. These scenarios included a review of the basic tenets of mechanical ventilation, acute respiratory distress syndrome (ARDS), pneumothorax, mucus plugging, and dynamic hyperinflation. Residents were divided into groups of 1 to 3 participants for each station. Participants were given a brief clinical description and expected to manage the ventilated patient for the first 10 minutes. Afterwards, intensivists had approximately 35 minutes for bedside debriefing. The debriefing included individualized assessment and feedback, summation of clinical teaching points, and review of a focused didactic PowerPoint presentation, tailored to each case.

### 2.6. Postintervention Evaluation

During the postintervention stage, participants individually underwent the same three scenarios as the preintervention stage, with faculty grading their performance using the same predetermined critical action checklists. This occurred ten days after the initial preintervention evaluation. At the conclusion of the scenarios, participants completed the postintervention cognitive multiple-choice test and confidence survey, as well as a postcurriculum survey.

### 2.7. Postcurriculum Survey

A postcurriculum survey was administered soliciting feedback on areas of strength and potential improvement of the curriculum using a 5-point Likert scale.

### 2.8. Data Analysis

Data were first imported into SPSS v22.0 software for analysis. Test results were summarized using mean (standard deviation), median, and range values for the percentage of correct answers at the pre- and postintervention study time points. The paired change in test results was determined as the postintervention test result minus the preintervention test result and then similarly summarized. The changes in test score were tested for median equality to zero using the nonparametric Wilcoxon signed rank test. Confidence was determined via 12 assessment questions measured on a Likert 1–5 ordinal scale at the pre- and postintervention study time points. Data were summarized using the aforementioned numeric measures of center of spread and the paired change data similarly tested for median equality to zero via Wilcoxon signed rank tests. Due to the potential for type I error rate inflation, *p* values were adjusted using Bonferroni adjustments to the *p* values to protect the overall type I error rate at 5%. Finally, for 3 of the 5 simulations, critical action performance was determined by expert review at the pre- and postintervention study time points. Paired dichotomous data (Yes/No) for each critical action was summarized with two-by-two bivariate frequency tables and tested for improvement in the discordant pairs with McNemar's tests. The paired changes, in total number of critical actions performed, were tested for median equality to zero using Wilcoxon signed rank tests for each of the 3 cases. All statistical testing was two-sided with *p* < 0.05 considered statistically significant.

## 3. Results

### 3.1. Demographics

Seventeen first-year residents participated in this study. The majority (64.7%, 11/17) were emergency medicine residents, while 23.5% (4/17) were general surgery residents, and 11.8% (2/17) were urology residents. All of the residents were present for the entirety of the boot camp.

### 3.2. Cognitive Knowledge Assessment

**Figure 2:**
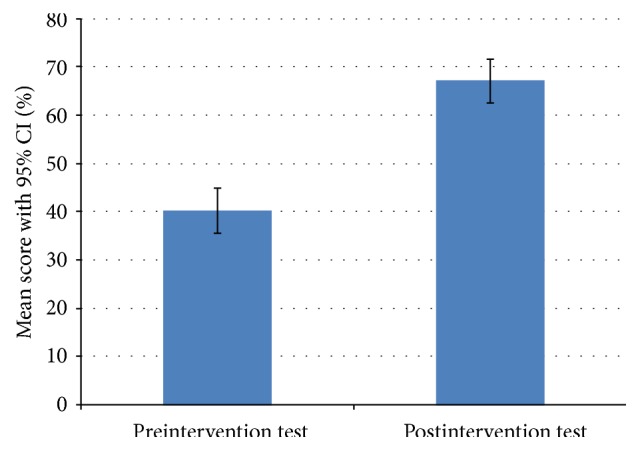
Pre- and postintervention knowledge assessment.

Cognitive knowledge between the identical pre- and postintervention multiple-choice tests increased significantly (Figure 2). The mean (SD) preintervention score was 40.3% (9.76%) with a median (range) score of 40% (25%–55%), and the postintervention mean (SD) was 67.1% (9.53%) with a median (range) score of 70% (50%–80%). The mean (SD) change in test score from before to after intervention was +26.8% (11.31%), with a median (range) score increase of 25% (5%–45%).

### 3.3. Clinical Performance and Critical Actions Assessment

**Figure 3:**
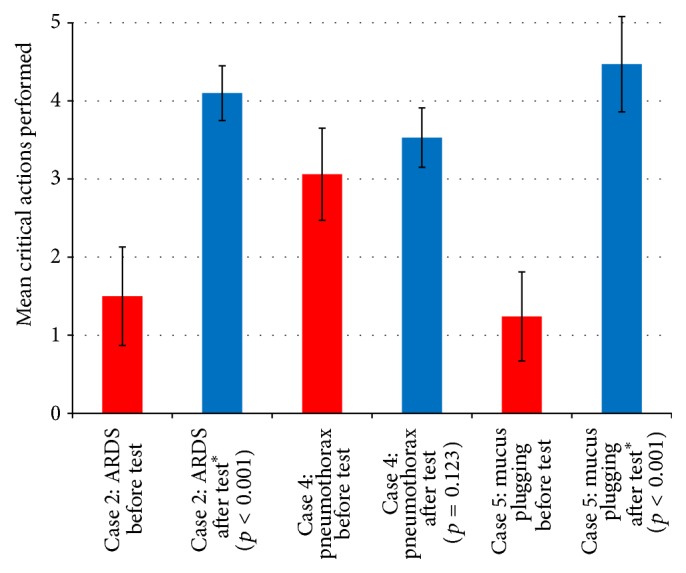
Critical actions assessment.

The increase in critical actions performed after intervention was significantly higher in the ARDS and mucus plugging cases but not significant in the pneumothorax case as shown in Figure 3.

The ARDS case had a significant increase in 4 of the 5 critical actions. Participants had a mean of 1.5 critical actions met during preintervention evaluation and 4.1 during postintervention evaluation (*p* < 0.001).

The atelectasis and mucus plugging case had a significant increase in 5 out of 7 critical actions. The mean preintervention score was 1.24 and the mean postintervention score was 4.47 (*p* < 0.001).

The pneumothorax case had no significant increase in critical actions. The mean preintervention score was 3.06 out of 7 critical actions and the mean postintervention score was 3.5 (*p* = 0.123).

There was no significant increase in the final critical action of all three cases. The final critical action was documentation of ventilator setting changes.

### 3.4. Confidence Assessment

Participants felt more confident with ventilator management based on their pre- and postintervention confidence surveys. This was supported by a statistically significant confidence increase for all questions, with a mean pretest score of 1.56 and a posttest score of 3.64 (*p* = 0.049). Mean confidence gain was 2.1 with a 95% confidence interval (1.6–2.6).

## 4. Discussion

This study demonstrates the feasibility and effectiveness of a boot camp curriculum for residents on the basics of mechanical ventilation. This curriculum resulted in increased competency, knowledge, and confidence of seventeen first-year residents. Using an integration of bedside instruction, supplemental study, and simulation-based training, residents were exposed to several pathologic conditions of mechanically ventilated patients, as well as appropriate assessment and management techniques.

A comprehensive ventilator management curriculum is long overdue and serves as a critically important patient safety initiative that may prevent iatrogenic morbidity. Residents often do not have uniform training in critical care before being expected to care for this patient population. Mismanagement of ventilated patients may result in significant morbidity and mortality [1]. It is no longer acceptable for residents and fellows to learn at the expense of the patient's health, especially in critical care environments. Medical education has subsequently evolved to include simulation as a way to educate, practice, and reinforce teaching points from cases that may otherwise only be seen rarely or in critically ill patients in the clinical setting.

During this curriculum, residents were exposed to several teaching methodologies to accommodate different styles of learning. Not only did residents asynchronously read about critical care topics, but they were able to interactively experience and troubleshoot high-acuity scenarios in a high-fidelity simulated environment. Residents were engaged in a supportive learning environment under direct supervision of an intensivist, who was able to provide personalized feedback and answer any immediate questions. Afterwards, they received a focused didactic PowerPoint presentation on the particular pathology they were managing. Familiarity with the equipment was improved by having the residents physically touch and manipulate the ventilators utilized in the ICU at our institution, helping to ameliorate any anxiety working with unfamiliar equipment. We postulate that these factors contributed to the statistically significant improvement in the various categories evaluated in this study.

If this curriculum motivated residents to seek out additional resources for their personal development, this could not be controlled for. Alternatively, we had no objective way of measuring if residents actually completed all provided readings. Our goal was to provide a novel and structured curriculum with dedicated time to improve the foundational knowledge in a skill set that typically did not have formal time set aside upon residents matriculation into residency. We did not intend to prove that any one method, such as simulation, was the sole contributing factor to improved performance but rather that the curriculum, as a whole, provided improved foundational knowledge.

The mechanically ventilated pneumothorax case was the sole scenario where there were no statistically significant increases in critical actions when analyzing pre- and postintervention evaluations. This case was unique in that definitive treatment required a procedure and ventilator management was an ancillary measure. All participants performed needle decompression or tube thoracostomy placement in the preintervention sessions, but only five participants increased FiO_2_ to maintain oxygenation, and only one increased tidal volume or rate to maintain adequate ventilation. We postulate that participants were more focused on the definitive management of tube thoracostomy versus adjusting ventilator parameters. We also speculate that medical school curricula more commonly provide instruction on management of pneumothoraces and related cardiorespiratory physiology in comparison to the topics of ARDS, atelectasis, and dynamic hyperinflation.

None of the three cases demonstrated a significant improvement in documenting ventilator changes. The importance of documentation was not covered in the supplemental reading material. Any emphasis of its importance would have been dependent on bedside instruction. Formal teaching on the necessity of proper documentation and its role within a patient safety context would likely reinforce appreciation and, therefore, compliance.

To date, there are very few studies in the medical education literature addressing mechanical ventilation curriculum for residents. We postulate that the reason that there are so few studies and curricula developed for simulation-based mechanical ventilation is likely due to the significant time and cost commitments required. This curriculum was financially expensive, time-consuming, and human-resource intensive. The significant financial cost arose from renting the lung simulators (approximately $5,000 USD). Our faculty and technicians needed eight hours of training on the lung simulators and a 6–25 hours' time commitment from each of the six different intensivists for the boot camp's execution. This does not include the time dedicated to the curriculum development, where a multidisciplinary team of specialists developed curricular goals and testing materials. This included determining the selected readings and critical action lists, as well as creating the cognitive multiple-choice questionnaire and confidence surveys. Deadlines for goals and objectives were established as a team and shared throughout the curriculum development over a period of approximately 8 months.

This study provides a curricular framework for other educators who desire to provide formal instruction to their residents on mechanical ventilation. Residency programs with an interest to develop and execute such a curriculum will improve their chances of success by pooling time and financial resources. Faculty members should work together to decide on goals and objectives early on in the process. Additionally, identifying how to finance renting necessary simulation equipment as well as how to divide up time for curricular and teaching responsibilities is paramount. Such cooperation will make the curriculum development less tedious and increase the likelihood of successfully executing such a curriculum. This curriculum only addressed pressure and volume control ventilation to focus on modes that residents will most commonly utilize during their training at our institution. Other modes of ventilation were not reviewed in order to prevent inundating residents with information unlikely to be used clinically. The expansion of this curriculum to include additional modes of ventilation and pathologies and the development of a longitudinal curriculum throughout residency training provide further opportunities for curriculum development, patient safety training, and research.

This curriculum was well received by participants as supported by feedback from a postcurriculum survey. Participants expressed that hands-on participation and bedside teaching were highly beneficial for their learning. They commented that some additional articles should be included in the reading assignment that provide a basic review of pulmonary physiology and an overview of mechanical ventilation, to establish a stronger foundational knowledge. Additionally, residents did not feel overwhelmed by the amount of material covered, as it was predetermined to only cover the two modes of ventilation.

*Limitations*. This was a small prospective pilot study from a community-teaching hospital. A randomized design with a control group of traditionally trained residents compared to residents completing this curriculum would have provided a more powerful study; however, residents and resources were limited. As the boot camp becomes further integrated into the residents' curriculum, additional medical specialties may be recruited to participate, which may also serve to provide subgroup analyses with greater power. Additionally, this study was done one time and repeating it over several years would both increase the number of residents in the study and check its reproducibility. In regard to data collection, a nonvalidated confidence survey, critical action checklists, and multiple-choice tests were utilized. We also did not survey participants to determine if they completed the provided reading materials or if they used other sources as supplementation.

## 5. Conclusion

A twelve-hour boot camp pilot study demonstrated significantly improved confidence, knowledge, and performance of first-year residents' ability to manage high-risk, low-frequency scenarios of mechanically ventilated patients. This study may serve as a model for other institutions that wish to implement a mechanical ventilation simulation curriculum within their training programs.
